# National guidance and district-level practices in the supervision of community health workers in South Africa: a qualitative study

**DOI:** 10.1186/s12960-019-0360-x

**Published:** 2019-04-03

**Authors:** Tumelo Assegaai, Helen Schneider

**Affiliations:** 1 School of Public Health, University of the Western Cape, Cape Town, South Africa; 2 University of the Western Cape/South African Medical Research Council Health Services to Systems Unit, University of the Western Cape, Cape Town, South Africa

**Keywords:** Community health workers, Ward-based outreach team, Support, Supervision, Document review

## Abstract

**Background:**

Supportive supervision is considered critical to community health worker programme performance, but there is relatively little understanding of how it can be sustainably done at scale. Supportive supervision is a holistic concept that encompasses three key functions: management (ensuring performance), education (promoting development) and support (responding to needs and problems). Drawing on the experiences of the ward-based outreach team (WBOT) strategy, South Africa’s national community health worker (CHW) programme, this paper explores and describes approaches to supportive supervision in policy and programme guidelines and how these are implemented in supervision practices in the North West Province, an early adopter of the WBOT strategy. Outreach teams typically consist of six CHWs plus a nurse outreach team leader (OTL).

**Methods:**

A qualitative, descriptive study that combined a document review of national policy and guidelines with key informant interviews in two districts of the North West Province was conducted. An overall WBOT policy statement and four guidelines on aspects of the strategy, spanning the period 2011–2017, were reviewed for statements on the three core facets of supervision outlined above. Eight focus group discussions, involving facility managers, team leaders and community health workers (total 40 respondents), purposively selected from four sub-districts in two districts, assessed local-level supervision practices. Alignment across policy and guidance documents and between policy/guidance and practice was examined.

**Findings:**

While all the official policy documents and guidelines reviewed acknowledged the need for supervision and support, these elements were inadequately developed and poorly aligned, both in terms of scope and in providing firm guidance on the supervision of WBOTs. The practices of supervision entailed a variety of reporting lines, while development and support processes were informal and often lacking, and teams poorly resourced. There was internal cohesion and support within teams amongst CHWs and between CHWs and OTLs. However, primary health care clinic managers, who were supposed to supervise the WBOTs, struggled to fulfil this role amidst the high workloads in facilities, and relationships between WBOTs and facility staff often remained strained.

**Conclusion:**

This study identified weaknesses in both the design and implementation of the supervision system of WBOTs. The lack of explicit, coherent and holistic guidance in policy and the failure to address constraints to supervision at local level undermine the performance and sustainability of the WBOT strategy in South Africa.

**Electronic supplementary material:**

The online version of this article (10.1186/s12960-019-0360-x) contains supplementary material, which is available to authorized users.

## Background

Evidence from countries around the world has shown that community health workers (CHWs) can contribute significantly to the efforts of improving the health status of populations, especially in countries with human resource for health crises [1, 2]. The benefits of CHW programmes in these countries include improved health outcomes and an expanded workforce.

However, there are still many challenges associated with CHW programmes, related particularly to their integration (or not) into health systems. Problems with CHW remuneration, training, role clarification, referral systems, information management and provision of supplies abound [1–3]. These challenges, combined with the fact that CHWs are equipped with limited skills and often work in remote and isolated areas, point to the need for supervision systems that not only monitor performance but also provide moral and other forms of support [4–6]. Reviews examining effective designs for CHW programmes have consistently found that the quality of supervision of CHWs affects the performance of programmes [7–13]. It also affects CHWs’ sense of belonging, morale, productivity, retention, respect and credibility with other stakeholders [7, 14–19]. Good supervision of CHWs, amongst other benefits, has the potential to improve and strengthen the relationships or interactions of CHWs with other health workers in the health system, resulting in improved trust and performance [20, 21]. Despite its importance, the literature provides little evidence of what a good supervision system for CHWs entails [1, 22, 23].

Supervision is a key component of human resource management and amongst a number of important strategies to improve health worker performance and health outcomes [6, 24]. Various definitions of health worker supervision are offered in the literature. Sennun et al. define it as “a process that involves monitoring work processes, understanding the causes of problems and providing possible solutions, as well as general management to improve operations, clinical direction, review guidelines, and providing approaches to effective service delivery, including patient safety, treatment and health promotion” [25]. This definition views supervision principally as a monitoring process that ensures compliance with standards and quality of care. It differs from definitions that explicitly consider supervision as not only a performance management and administrative tool, but also as a mechanism of personal and developmental support to health workers. One of the broader definitions to supervision would be “process that promotes quality at all levels of the health system by strengthening relationships within the system, focusing on the identification and resolution of problems, and helping to optimize the allocation of resources” [6]. Another is “the provision of monitoring, guidance and feedback on matters of personal, professional and educational development in the context of the doctor’s care of patients” [26]. In the South African context, the need for a supportive environment is echoed in the country’s Human Resource for Health Strategy. The strategy states that “the key role of the leadership of the health sector at all levels is to ensure a healthcare environment in which the health workforce is valued and supported and has the opportunity to develop while providing high quality care” [27].

Holistic definitions outline three basic functions served by supervision: management/administrative, educational/developmental and support [26, 28]. The management function is concerned with ensuring compliance with organisational standards and policies, the developmental function seeks to improve knowledge and skills to perform, and the support function addresses morale, motivation and job satisfaction. Peach and Horner [28] separate these functions into the “production” and “people” aspects of supervision; production is centred on management functions and “people” on education and support functions. They argue that improving health outcomes (production) and resource development (people) cannot be addressed independently, but are rather complementary and equally important.

Community health workers are not a new phenomenon in South Africa. Over the years, CHWs have played a significant role in the health sector in a wide variety of areas such as maternal and child health, HIV, TB and other chronic conditions [29–32]. In 2011, The National Department of Health (NDoH) in South Africa introduced the Re-engineering of Primary Health Care (RPHC) strategy as one of a set of health system reforms to address system weaknesses that resulted in the country only partially achieving the Millennium Development Goals (MDG) related to maternal, child and infant mortality; HIV; and TB [33, 34]. The RPHC strategy recommended, amongst a number of reforms, the Ward-based PHC outreach team strategy to strengthen health prevention and promotion, identify individuals and families at high risk and build links between households and health care facilities. The ward-based outreach teams (WBOT) constitute South Africa’s national CHW programme and feature in key national policy platforms including the National Development Plan 2030 [35] and the National Health Insurance White Paper [36]. The WBOT strategy represents the latest and most significant in a line of policy initiatives over the last decade to shape the community-based sector.

A ward-based PHC outreach team (WBOT) is comprised of a nurse as the outreach team leader (OTL) and an average of six CHWs. The team is attached to a facility, operates within a municipal ward and provides promotive and preventive services to individuals at household level. Training for CHWs is standardised with official tools outlining the functions of the team leaders, CHWs, facility managers and other managers at the district, province and national levels. National guidelines, policy and training documents were developed for the WBOT strategy, specifying roles and functions for both CHWs and OTLs [37, 38], which were to be implemented at provincial and district level.

This article describes the extent to which the national policy and training documents related to WBOTs in South Africa provide guidance on supervision processes; how these documents conceptualise supervision; and how they balance the production and people components of supervision. The article then explores how those involved in implementing the WBOT strategy perceive the current supervision practice versus prescribed policy and training documents. This study aims to contribute towards understanding of the design of supervision strategies, and their alignment with the implementation of support and supervision processes in CHW programmes. The study was based in two districts of the North West Province, an early adopter of the programme. The province started implementation immediately after the NDoH announced the programme in 2011, with pilot teams in all 19 sub-districts across the province by 2012. By December 2015, 72.6% of the 382 wards in the province reported functional teams, compared to an average of 36.4% for the country as a whole [39].

## Methods

A descriptive qualitative study of policy and practices related to supervision of WBOTs in two districts of the North West Province was conducted.

To describe the policy on WBOT supervision, all NDoH guidelines, policy and training documents related to the WBOTs and available in the public domain since the inception of the WBOT programme were sourced. To explore practices, focus group discussions were held with facility managers, team leaders and community health workers involved in the immediate supervision system of WBOTs in two districts of the North West Province.

Focus group discussions were conducted in one sub-district in each district. From each sub-district, one older and one recently established outreach team and their associated PHC facility managers were purposefully sampled. The four ward-based outreach teams were purposefully sampled, in consultation with sub-district managers, as being typical examples of functioning WBOTs established in the earlier and later phases of the programme, and for their knowledge and experience, and therefore, potential as information rich cases.

### Document review

The document review was conducted on all NDOH policy and training documents, which contained any text relating to supervision or support of the WBOT programme. The policy framework and strategy for WBOT document was sourced from NDoH soon after its distribution. The remaining documents were obtained from the provincial office responsible for overseeing the programme. The district confirmed that they used most of them as reference documents. The documents include a set of guidelines issued in the inception stages of the programme (2011), three guides for CHWs (2014), team leaders (2012), and middle to top managers (2012), respectively, and a recent policy framework (2017). The documents are listed in Table 1 in chronological order of publication.

**Table 1:** Documents reviewed

Title of document (short title)	Purpose	Year of publication
Provincial guidelines for the implementation of the three streams of PHC Re-engineering (Toolkit)	Provincial guidelines and toolkit for implementation of the WBOT programme	2011
CHW participant guide—phase 1 (CHW manual)	Accredited training guide for CHWs	(First version 2011) 2014
Ward-based PHC outreach team leader orientation programme learner guide (Team Leader Guide)	Orientation guide for team leader on their roles	2012
Ward-based PHC outreach teams management information (Management Guide)	Middle and top management overview of WBOTs’ value, purpose, roles and responsibilities.	2012
Policy framework and strategy for ward based primary healthcare outreach teams (Policy)	A framework to improve WBOTs’ working conditions and standardise their scope of work and application across the provinces.	2017

All the text related to supervision and support for CHWs and the WBOTs was extracted from the documents and entered into an excel spreadsheet. The text was organised along the three domains of supportive supervision (management, development, support) that emerged from the literature. Within each of the three domains, themes and sub-themes and the specific elements were inductively coded based on the material in the documents.

Both authors agreed on the framework for the analysis and read the documents. The first author (TA) did the coding, which was then discussed and validated with the second author (HS).

### Focus group discussions

A semi-structured interview guide with open-ended questions on the supervision of WBOTs was used to conduct focus group discussions (FGDs) with a total of 40 respondents (Table 2).

**Table 2:** Focus groups participants

Level	Number of people	Groups
Facility managers	10	2
Team leaders	12	2
CHWs	18	4

Respondents were provided with information sheets to familiarise themselves with the research topic and given an opportunity to ask questions. They gave written consent to participate in the study and were made aware of their right to withdraw from the study at any time. FGDs were conducted with the three categories—facility managers, team leaders and CHWs—as separate groups to avoid power relations arising from professional status and hierarchies inhibiting participation. A semi-structured FGD guide loosely structured the discussion, allowing for probing for more information and seeking clarification where necessary. The FGDs were conducted by the first author (TA) and took place at respondents’ place of work as chosen by the sub-districts. All the interviews were conducted in English, including the CHWs, all of whom have at least secondary level schooling and attend training programmes in English. The interviews were audio recorded, transcribed and coded using the ATLAS.ti 8 (ATLAS.ti Scientific Software Development GmbH, Berlin).

Analysis of FGDs was done using the thematic content analysis approach [40]. The researchers read all the transcripts to familiarise themselves with the text, then identified codes, categorised the codes and developed themes and sub-themes that emerged from the text based on the three basic functions of supervision (management, development and support). The researchers then analysed the strengths, weaknesses, gaps and alignment between the official positions on supervision (from the document review) with practices (from the interviews).

The FGDs were conducted as part of a longer association of the authors with the WBOTs in the North West Province, in both support/technical (TA) and research (TA and HS) capacities. The trustworthiness of the study was thus enhanced by these well-established local relationships, shaping the depth and quality of FGDs, and the ability to draw on wider contextual and tacit knowledge in the analysis.

### Findings

#### Policy and guidelines

The Toolkit is the first document that was distributed at the beginning of the programme and is widely used as a reference guide for implementation. However, this document remains in draft format and is yet to be revised or issued as a final document. The Policy document is the most recent and most significant of the documents, but lists supervision functions in very summary terms.

Table 3 summarises the official guidance on supervision by document source and theme—management, development and support. Management was further categorised into sub-themes of line authority, performance management and provision of resources. The plus sign (+) denotes the degree of emphasis placed on the particular function in each document. These summary judgements were based on the full text extracted from the documents that talks to supervision (provided in Additional file 1).

**Table 3:** Coverage of functions (+) per theme across the documents

Document	Management			Development	Support
	Line authority	Performance evaluation	Resources		
Toolkit	+++	+++			++
CHW manual	++++	+	+	+	+
Team Leader Guide	++++	++	+	+	+++
Management Guide	++	+++	+	+	++
Policy	+++	+++	++	+	+

The line authority sub-theme includes the following functions as captured from the documents: the recruitment of team leaders, ideal candidates for team leader positions, CHWs supervisor and team leader supervisor. Only two of the guides—the CHW Manual and Team Leader Guide—cover all these functions comprehensively. According to the documents, districts and sub-districts appoint team leaders and facility managers supervise and participate in the recruitment of team leaders. The team leader’s scope of work requires a professional nurse (4-year qualification), but with a shortage of this cadre, the new Policy document encourages provinces to “Identify mechanisms for each facility to assess current staff vis-a-vis new PHC structure – particularly with respect to who will supervise the outreach team” [39]*.* There is consensus across documents that team leaders are to supervise CHWs and oversee activities of the team and that CHWs report to the facility manager through their team leaders.

The performance evaluation sub-theme functions include how to monitor, record and report on performance, and the designation of responsibility for these functions to facility managers and team leaders. As with the line responsibilities, these functions are addressed in all the documents, bar the CHW Manual, which only mentions that the team leaders manage the performance of team members with no further details. The Team Leader Guide goes further to include performance evaluations forms for CHWs, developed by supporting partners as part of the monitoring and evaluation (M&E) system tools at the inception of the WBOT programme. However, when NDoH adopted the M&E system for WBOTs, the performance evaluation forms were not officially endorsed for use by teams, and none of the other documents reviewed refer to these forms.

The resources sub-theme includes the provision and management of basic resources and availability of physical space for storage of records and team meetings. Basic resources for service delivery include transport, stipends, basic clinical supplies and stationery for recording keeping and reporting. The Policy document states that the provincial Department of Health will fund the programme and make available resources for the teams and that it is the responsibility of the facility and sub-district managers to supply and manage these resources. It further mentions that the department will ensure availability of space for WBOTs through the Ideal Clinic programme, a national clinic accreditation programme. The remaining documents refer to resources in either passing or not at all. In none of the documents is there a specific list of items to be supplied.

The development theme relates to the level of guidance provided in the documents on capacity building for WBOTs members and their supervisors. There is formal training for CHWs and orientation for team leaders, facility managers and middle managers to support the programme. The CHW manual is the South African Qualifications Authority (SAQA’s) accredited curriculum for the first phase of the formal training. The documents mention that, beyond the formal CHW training, supervision of WBOTs includes training, mentoring and coaching of CHWs. This capacity building is to be achieved through induction, skills development, clinical guidance and technical support in the form of in-service trainings and workshops. According to the CHW Manual, Team Leader Guide and the Management Guide, the team leader is responsible for CHWs’ capacity development. However, this is not categorised by format, frequency and content. The CHW Manual identifies the health promoter as a source of technical support on health promotion but also provides no further details. The Policy document simply mentions that the department will confirm the training content and method to build the required capacity for CHWs and the development and maintenance of a capacity building system at district level. In general, basic training is well established, but further development post in-service is only superficially acknowledged.

Except for the Team Leader Guide, the documents provide some guidance on how supervisors need to support WBOT members, but do so in very limited terms. The Team Leader Guide provides more details around mentoring and coaching of the WBOT members.

In sum, the documents reviewed provide considerable detail on the management functions of supervision, but much less on development and support, the two other crucial pillars of supportive supervision. All the documents acknowledge the need for supervision and outline basic reporting lines. One of the objectives in the Policy document seeks to “ensure adequate supervision and support for CHWs as well as for team leaders” but provides no elaboration. Neither the Toolkit nor the Policy spells out a comprehensive approach to supervision, support and line authority functions. Rather, decision-making is delegated to sub-national levels. For example, the Toolkit refers to “Setting up supervision, reporting and monitoring systems for outreach teams through consultations with heads of facilities (through sub-district/ district-level meetings)”. The Policy document refers to, as one of the key responsibilities for the province, “approving the implementation plan in the districts…”, and for the district to “develop an implementation plan…”.

The training documents, on the other hand, provide considerably more detail on the procedures and style of supervisory relationships. Both the Team Leader Guide and the Management Guide were piloted in the North West Province and distributed through workshops at the beginning of the programme, and the team leaders who were in pilot WBOTs at the time were oriented on the contents. However, the induction workshops were subsequently discontinued and the Team Leader Guide document was then distributed as part of the team leader package, where its status remains semi-official. The CHW manual remains in use as part of the first part of the formal training for CHWs.

While the documents reviewed refer to supervision in various places, currently, there is no standalone, overarching and coherent framework or document for the supervision of CHWs and WBOTs. Moreover, most of the documentation, which exists, although widely available and referenced, has uncertain status.

### Supervision practices

#### Management

In establishing the line authority function of WBOTs, the North West, as other provinces, struggled to attract professional nurses as team leaders to rural areas where most of the WBOTs are based. As a result, the province sought to recruit professional nurses from facilities and retired nurses to work as team leaders. Team leaders were recruited in a variety of ways, most commonly volunteering to take on the role.

… I depend on walk-ins (manager).

… so we volunteered (professional nurse, district 1).

So I was just requested [to be a team leader] (professional nurse, district 1).

I heard over the radio that there was an advertisement… So I went to the district office to find out about that … it was confirmed and then we had to do some applications and … we were called for an interview. (retired nurse, district 2).

In most areas, facility managers were tasked with supervising the outreach teams. It would appear that the department did not explain the WBOTs’ scope of work to facility managers “we didn’t know what was expected of us” (facility manager, district 2). The facility managers were also not involved in the recruitment of team leaders. As one facility manager responded, “I was just told… [I was] not part of the selection” (facility manager, district 1). Facility managers mentioned things such as “supervise, discipline, in-service training, provide resources” (facility manager, district 2), to highlight what they thought their role was towards outreach teams. However, as one facility manager expressed, there was uncertainty on what this role really entailed in practice “… we are not told how far you should go with the management of the team leader” (facility manager, district 1). In some areas, districts delegated professional nurses as “focal persons” at sub-district and district levels to coordinate and oversee the WBOTs programme, who sometimes also directly supervised the team leaders.

As indicated, performance evaluation forms to enable team leader to monitor and review the performance of CHWs were developed and distributed during the inception phases of the programme in the North-West. Although the FGDs suggested that there was some form of unofficial evaluation occurring between team leaders and CHWs, as one CHW explained, “…[the team leader] checks that I present myself well and that I fill the forms correctly” (old CHW, district 1), the performance evaluation forms were never made official and most team leaders were not oriented on them. There was no formal performance review system for team leaders and WBOTs as a whole. This was compounded by the fact that CHWs and retired nurses (as team leaders) were contracted on a short-term basis with no performance agreement. As a result, facilities felt they had no control over the functions of team leaders and WBOTs, even if informal monitoring took place. As one operation manager indicated “[there is] no measuring system where we measure their progress and performance.” (facility manager, district 2). Some facilities also reported holding meetings with the WBOTs to update each other on achievements and challenges within the communities.

At the beginning of the programme, the department provided the majority of the CHWs with kit bags as part of their phase 1 training. These bags had basic supplies such as bandages, gloves, and condoms. The districts instructed facilities to replenish the supplies of WBOTs reporting to them on an ongoing basis. However, CHWs indicated that the supplies were limited and not provided regularly. Some facility managers made mention that they provided resources such as gloves and nappies to outreach teams. However, not all CHWs concurred, as one CHW stated, “the facility will say it’s not their job to give us [supplies]” (old CHW, district 1). All teams indicated that they did not have space to work and had to improvise with solutions to do their work and keep records safe. As explained by one team leader “I don’t have any space for my records. I keep them in my car…” (team leader, district1).

There is shortage of transport in the province, and in instances where wards are vast, households are hard to reach on foot. As a result, the province decided to allow team leaders who had vehicles to use them for WBOTs support and claim for up to 500 km travelled per month. However, team leaders indicated that there were problems with this arrangement as they would sometimes also be expected to transport supplies for the facilities such as medicines and administration materials, “… we are a shuttle service” (team leader, district2).

#### Development

The basic training of CHWs is provided through accredited Regional Training Centres located at district level. Trainers include maternal and child programme coordinators, team leaders and professional nurses, who are not necessarily team leaders. Team leaders are encouraged to attend CHWs trainings to familiarise themselves with the curriculum and observe how CHWs perform in the training. In some instances, team leaders are also trainers. As indicated earlier, in the inception phases of the programme, the department provided a non-compulsory 5-day orientation workshop for team leaders in the province. However, team leaders recruited beyond the pilot phase were not offered these workshops.

Team leaders regarded it as their responsibility to provide CHWs with in-service training to improve clinical and technical skills and appeared motivated to improve the capacity of CHWs. As one team leader explained, “it is our responsibility to give CHWs in-service training, guide them how to deal with communities and whenever they encounter challenges they are encouraged to consult us” (team leader, district 2).

#### Support

Team leaders were also reported to have good relations with CHWs. As explained by one CHW, “Our relationship with our team leader is excellent … [when] we want the team leader to go with us to make a follow up… she comes. The presence of team leaders at household level allows the clients to be more receptive to the service and makes the work easier” (new CHW, district 1).

Outreach teams interacted with facility staff, but WBOT members generally felt that facility managers did not understand the role of the teams. Facility managers were described as putting pressure on teams to assist in the facilities. As one team leader narrated, “the facility manager usually says, we have a shortage … go to another [consulting room] and assist” (team leader, district 1). As a result, team leaders felt constrained in supporting CHWs in the communities. However, not all facility managers were described in negative terms. Some understood their role as supportive, as expressed by one facility manager, “the role of the facility manager is to empower them… check their challenges and then address them” (facility manager, district 2).

Relationships between WBOTs and other facility staff varied. There were teams where relations had evolved positively, as one CHW recalled, “Firstly, the nurses from the facility were treating us badly. Now they are much better, they know our role” (new CHW, district1).

Others felt they were poorly treated:

If you tell them that I have to go out and assess the CHWs, they will say we are just gallivanting in the location, you are not doing anything. (team leader, district 2).

Our relationship is very, very poor in the clinic, very poor (old CHW, district 1).

Most sub-districts in the province have programme coordinators that are responsible for different programmes such as maternal and child health and chronic disease care. However, there was no indication that there was any interaction between programme coordinators and outreach teams.

In sum, in the absence of a clear supervision framework, teams and facilities functioned in an ad hoc manner that best suited them in the delivery of services. In practice, there was a variety of reporting lines, development and support processes were informal and often lacking, and teams poorly resourced. There was internal cohesion and support within teams. However, facility managers struggled to supervise the teams amidst high workloads in facilities, and relationships between WBOTs and facilities often remained strained.

## Discussion

The WBOT programme plays a critical role in extending PHC services to community and household level and making health accessible in terms of distance and information [41]. Community health workers render services at household level, with limited training, resources and support. It is therefore important that CHWs are well trained, adequately supervised and supported to withstand challenges and deliver quality services [42, 43]. Although studies argue that adequate support and supervision are essential for the success and performance of CHW programmes at scale, the development of supervision systems for CHWs in policy and practice remains a challenge globally [18, 44–46].

While all the official policy documents and guidelines reviewed acknowledge the need for supervision and support, they are inadequately developed both in terms of scope and in providing firm guidance on supervision of WBOTs. Moreover, texts on supervision are not standardised, neither do they cross-reference each other, with some aspects present in some documents, but absent in others. In relation to the basic functions of supervision, the documents generally had more details around management and less so on development, and to some degree, also on support. The absence of a coherent framework for supervision of CHWs/WBOTs, and the misalignment and lack of details on supervision observed in these documents are likely to impact on the effectiveness of WBOTs [47].

In the absence of aligned and mutually reinforcing policy documents and a holistic supervision framework, supervision and support of WBOTs is poorly done. Policy and training documents outline a line of authority between CHWs, team leaders and facility managers. However, the dearth of professional nurses affects the recruitment of team leaders, and as a result, the WBOT programme is seen as an added responsibility and burden [32, 48].

Team leaders in their role as supervisors of CHWs are generally regarded as good supporters [49]. Facility managers typically supervise team leaders, but their support is often perceived to be lacking. A study in Uganda found that supportive supervision and relationships between CHWs and facilities affected performance of the programme [44]. Problematic relationships between facilities and CHWs are well described in literature [50, 51]. The root of the problem may be inadequate integration of CHW programmes into the health system; overburdened and poorly resourced facilities; conflicting interests between facilities and CHWs; weaknesses in the support and supervision of facilities themselves; and limited participation by stakeholders in the design and decision-making of the CHW programme [7, 52–55]. There is a need for further research to understand factors associated with strained relationships between facilities and CHWs.

Performance management has been defined as a process that is used to measure and improve the performance of workers in order to improve the performance of the organisation [56–58]. Despite efforts to improve performance of the programme through building capacity of CHWs [15], performance management for both CHWs and team leaders is unofficial, and the process is often unrecorded.

The list or package of basic resources WBOT members need to perform their functions is not explicit in the policy documents and WBOTs had limited basic resources and physical space [48]. A South African study looking at factors affecting access to care found that a lack of resource acted as a barrier in providing services for CHWs [59]. Supervision can mitigate the supply of resources for CHWs [5].

There is formal basic training for CHWs in the WBOT programme, but the induction and in-service training for CHWs is not formalised and organised [60]. Supervision thus affects the likely impact of CHW training on performance [46].

The national frameworks reviewed substantively shaped how the North West Province approached the supervision of WBOTs and the findings in this province are likely to be mirrored in other provinces. Although provinces are required to develop implementations plans where adaptations may be introduced within the broad framework, in practice, at the time of this research, the national policy documents were being implemented without much provincial and local adaptations. Nonetheless, the day-to-day experiences of supervision largely depend on the nature of local leadership and context from districts to facilities, and in turn, this is likely to result in variations across provinces and districts.

## Conclusion

This study identified weaknesses in both the design and implementation of the supervision system of WBOTs. The lack of explicit and coherent guidance in policy, and the failure to address constraints to supervision at local level, undermines the performance and sustainability of the WBOT strategy in South Africa. The study highlights the need for holistic conceptualisations of the supportive supervision function in policies on CHW programmes, and the importance of recognising the key facilitators and barriers to local implementation. In particular, CHW programme designs based on teams (peer support) and dedicated professionals to support them (such as outreach team leaders) enable supportive supervision. Conversely, PHC facility managers cannot be assumed to be willing and capable supervisors of CHWs and need to be adequately prepared and supported to fulfil this role.

## Additional file

Additional file 1: A full text extracted from the documents that talks to supervision. (XLSX 14 kb)

## Abbreviations

CHW manual: CHW participant guide—phase 1
CHW: Community health worker
FGDs: Focus group discussions
M&E: Monitoring and evaluation
Management Guide: Ward-based PHC outreach teams management information
MDG: Millennium Development Goals
NDoH: National Department of Health
Policy: Policy framework and strategy for ward-based primary healthcare outreach teams
RPHC: Re-engineering of Primary Health Care
Team Leader Guide: Ward-based PHC outreach team leader orientation programme learner guide
Toolkit: Provincial guidelines for the Implementation of the Three streams of PHC Re-engineering
WBOT: Ward-based PHC outreach teams
